# Antifungal wound penetration of amphotericin and voriconazole in combat-related injuries: case report

**DOI:** 10.1186/s12879-015-0918-8

**Published:** 2015-04-15

**Authors:** Kevin S Akers, Matthew P Rowan, Krista L Niece, John C Graybill, Katrin Mende, Kevin K Chung, Clinton K Murray

**Affiliations:** United States Army Institute of Surgical Research, 3698 Chambers Pass, JBSA Fort Sam Houston, TX 78234 USA; Department of Medicine, Infectious Disease Service, San Antonio Military Medical Center, 3698 Chambers Pass, JBSA Fort Sam Houston, TX 78234 USA; Infectious Disease Clinical Research Program, Uniformed Services University of the Health Sciences, Bethesda, MD 20814 USA; Department of Medicine, Uniformed Services University of the Health Sciences, Bethesda, MD 20814 USA

**Keywords:** Invasive fungal infection, Pharmacokinetics, Negative-Pressure Wound Therapy, NPWT, Effluent

## Abstract

**Background:**

Survivors of combat trauma can have long and challenging recoveries, which may be complicated by infection. Invasive fungal infections are a rare but serious complication with limited treatment options. Currently, aggressive surgical debridement is the standard of care, with antifungal agents used adjunctively with uncertain efficacy. Anecdotal evidence suggests that antifungal agents may be ineffective in the absence of surgical debridement, and studies have yet to correlate antifungal concentrations in plasma and wounds.

**Case presentation:**

Here we report the systemic pharmacokinetics and wound effluent antifungal concentrations of five wounds from two male patients, aged 28 and 30 years old who sustained combat-related blast injuries in southern Afghanistan, with proven or possible invasive fungal infection. Our data demonstrate that while voriconazole sufficiently penetrated the wound resulting in detectable effluent levels, free amphotericin B (unbound to plasma) was not present in wound effluent despite sufficient concentrations in circulating plasma. In addition, considerable between-patient and within-patient variability was observed in antifungal pharmacokinetic parameters.

**Conclusion:**

These data highlight the need for further studies evaluating wound penetration of commonly used antifungals and the role for therapeutic drug monitoring in providing optimal care for critically ill and injured war fighters.

## Background

A significant number of combat-related casualties have resulted from the ongoing military operations in Iraq and Afghanistan [1]. Improvements in tactical combat casualty care have led to increased survival rates [2,3], resulting in prolonged survival of critically ill and injured patients who experience complex treatment courses, often with complications. Invasive fungal infection is a major complication that affects patients regardless of immune status [4], with reported risk factors including injury while serving in riparian environments of southern Afghanistan [5], dismounted blast injury resulting in polytrauma (severe lower extremity and groin trauma from stepping on an improvised explosive device), and massive blood transfusion [6]. Inoculation of soil-dwelling molds into wounds is proposed to occur at the time of injury, with subsequent growth of vegetative mold structures within human tissue. Fittingly, invasive fungal infection has been recently documented in patients that sustained wounds contaminated with wood, soil, and gravel following a major tornado [7]. Diagnosis is often delayed by the need for specimen collection, limited microbiological assays in austere field hospitals, histopathological confirmation, and positive fungal culture. Thus, the median time from infection to diagnosis is 10 days [6]. Treatment is primarily surgical debridement, as the efficacy of antifungal agents remains unclear [8,9]. In clinical practice, antifungal agents are anecdotally observed to be ineffective in halting the progression of invasive infection despite what is perceived to be adequate surgical debridement. As a result, aggressive debridements into clinically viable tissue are often necessary, resulting in severely disabling procedures such as extremity amputation, hip disarticulation, or even hemipelvectomy.

In lieu of antifungal therapies sufficiently potent as to eliminate the burden of life-saving but disabling surgeries, optimizing the dose of currently available antifungal agents might help maximize clinical recovery while limiting toxic side effects. Furthermore, little is known about tissue penetration of antimicrobials, including antifungals, and whether plasma concentrations are correlated to wound penetration. Negative pressure wound therapy (NPWT), frequently used in the care of wound infections, affords an opportunity to collect wound effluent for evaluation. Previous studies have examined wound effluent for the presence of bacteria [10], inflammatory cytokines [11-15], and other protein components [16] but the presence of antimicrobials in wound effluent has not been examined. To gain insight into the penetration of clinically useful antimicrobial agents across wounds under negative pressure, we undertook two prospective observational studies of antimicrobials, including antifungals, in patients with trauma-related wounds. Here we report the systemic pharmacokinetics (PK) and effluent antifungal concentrations of five wounds in two blast-injured combat casualties with proven or possible invasive fungal infection.

## Case presentation

This study was conducted under a protocol reviewed and approved by the U.S. Army Medical Research and Materiel Command Institutional Review Board and in accordance with approved protocols BAMC C.2013.095 and H-09-059. Two patients receiving antifungal therapy for proven or possible invasive mold infection were enrolled with informed consent into institutional review board-approved observational pharmacokinetic (PK) studies examining the adequacy of antimicrobial dosing in critically ill patients, or in the context of military wound infections. At the time of initial PK steady-state sampling, both patients were receiving liposomal amphotericin B (L-AmB) with one also receiving voriconazole at recommended maintenance doses (5 mg/kg and 4 mg/kg, respectively). Peripheral blood samples were obtained at time points spanning the dose interval for each agent. Simultaneously, wound effluent was collected over 24 hours (the dose interval for L-AmB) from available sites treated with NPWT (Wound V.A.C., KCI, San Antonio, TX) into canisters lacking the congealing agent. Wound effluent was recovered from canisters by syringe aspiration for volumetric measurement and determination of antifungal concentration.

Total and free concentrations of amphotericin B and voriconazole were determined in plasma and wound effluent using high-performance liquid chromatography (HPLC). Data was collected on a Dionex UltiMate 3000 instrument (ThermoFisher, Waltham, MA) equipped with an HPG 3400SD binary pump, WPS autosampler, and DAD-3000 UV-visible detector. For amphotericin B, analysis was conducted as previously described [17] with slight modifications. The mobile phase consisted of 10 mM acetate buffer (pH 4.0) and acetonitrile, delivered isocratically at a 63:37 ratio and a flow rate of 0.6 mL/min [17]. The stationary phase was a Phenomenex Luna C18(2) 150x4.6 mm column (100 Å pore size, 5 μm particle size) preceded by a 10 mm guard column with matching chemistry. Seven calibration standards, 150 μL each, were prepared containing 0.1-200 μg/mL amphotericin B on a free drug basis (deoxycholate, Sigma, St. Louis, MO) and 50 μL of 25 μg/mL natamycin as an internal standard to correct for losses during sample preparation. One mL of acetonitrile:acetic acid (9:1) was added to each sample, which was then vortexed briefly and stored at room temperature in darkness for 1 hour. Each sample was centrifuged at 10,000 × *g* for 10 min to precipitate protein from the sample, after which the supernatant was loaded onto the HPLC. Detection was by UV at 405 nm for amphotericin B and 303 nm for natamycin. Injection volume was 50 μL. Retention times were 10 min for amphotericin B and 4.5 min for natamycin. The calibration curve was constructed using the peak area ratio of the analyte (amphotericin B) and the internal standard (natamycin). To determine total drug levels in patient samples, 50 μL of 25 μg/mL natamycin was added to 150 μL of plasma or effluent and the same preparation and analysis procedures were followed.

Voriconazole was identified by HPLC as previously described [18,19]. The mobile phase consisted of 10 mM phosphate buffer (pH 7.0) and acetonitrile, delivered in a gradient (20-50% acetonitrile over 10 minutes) at a flow rate of 1 mL/min. The stationary phase was a Phenomenex Luna Phenyl-Hexyl 150x4.6 mm column (100 Å pore size, 5 μm particle size) column preceded by a 10 mm guard column with matching chemistry. Six calibration standards were prepared containing 1–50 μg/mL voriconazole (SelleckChem, Houston, TX) and 1 μL of 50 μg/mL itraconazole as an internal standard to correct for losses during sample preparation. Sample cleanup was accomplished by protein precipitation with 400 μL methanol followed by centrifugation at 10,000 × *g* for 10 min. The supernatant was dried under air (15–20 L/min) and reconstituted in 200 μL MeOH:phosphate buffer (1:1). Detection was by UV at 260 nm for both voriconazole and itraconazole. Injection volume was 25 μL. Retention times were 3.6 min for voriconazole and 9.8 min for itraconazole. To determine total drug levels in patient samples, 1 μL of itraconazole (50 μg/mL in methanol) was added to 200 μL of each plasma or effluent sample and the same preparation and analysis procedures were followed.

Free (protein unbound) levels of both drugs in plasma and effluent were determined using ultrafiltration units as previously described [20-22]. Briefly, 300 μL of plasma or effluent was loaded into each cartridge and samples were centrifuged at 1,500 × *g* for 30 min. The filtrate was assayed by HPLC as described above and the results were volume-corrected to find the concentration of unbound drug for each sample.

From the resulting plasma time-concentration curves, PK parameter estimates were determined by non-compartmental analysis using standard WinNonLin v6.3 (Pharsight Inc., Sunnyvale, CA). The adequacy of antifungal concentrations in wound effluent to suppress mold infections were interpreted using minimum inhibitory concentrations (MICs) of 0.5 μg/mL for amphotericin B against Mucorales molds and 1.0 μg/mL for voriconazole against *Aspergillus* spp. [23,24], the lowest concentrations at which growth of these organisms are reliably inhibited *in vitro*. Potential drug interactions were evaluated using the Lexi-Interact™ Online software (Lexicomp, Inc., Hudson, OH).

### Patient 1

**Figure 1:**
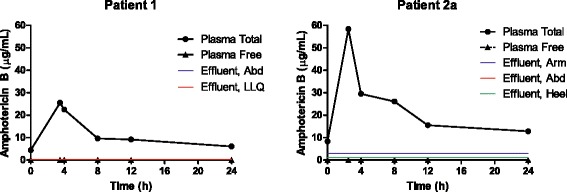
Amphotericin B concentrations in plasma and wound effluent in two patients with 5 wounds. Abd, abdomen; LLQ, left lower quadrant.

A 30 year-old male suffered a complex blast injury as a result of a dismounted improvised explosive device blast in southern Afghanistan, resulting in polytrauma including bilateral above-the-knee amputations, perineal/gluteal avulsion and 20% total body surface area burns to the posterior thighs and back. Initial resuscitation and damage control surgery was performed in Afghanistan, followed by medical evacuation on post-injury day 3 to Landstuhl Regional Medical Center (LRMC) in Germany. At LRMC, tissue samples of the lower extremity amputation sites and gluteal wounds were collected as part of clinical care for early detection of invasive fungal infection in accordance with the LRMC “blast protocol” [8], demonstrating angioinvasive non-septate branching hyphae diagnostic of a proven invasive fungal infection by a Mucorales mold [9]. Repeated surgical debridements performed at the discretion of the clinical care team with tissue fungal cultures (post-injury days 3 to 7) resulted in recovery of *Aspergillus flavus*, *A. terreus*, *Fusarium* sp. and an unidentified Mucorales mold. Following transfer to Brooke Army Medical Center, subsequent tissue fungal cultures from debridements recovered *Saksenea erythrospora* on 10 different occasions between post-injury days 12 and 46, with non-septate branching hyphae observed but not cultured on 5 additional soft tissue specimens collected on 3 separate days. Additionally, *Fusarium* sp. was recovered on post-injury day 59. Plasma and effluent sampling was performed on post-injury day 18 (Patient 1, Table 1, antifungal treatment day 15) following a once-daily dose of L-AmB (5 mg/kg IV), with effluent recovered from two abdominal NPWT sites (midline and left lower quadrant, Figure 1). While the plasma peak and trough concentrations were 25.5 μg/mL and 6.1 μg/mL, respectively (Table 1), total amphotericin B in the wound effluent was 0.2-0.3 μg/mL and free amphotericin B levels were undetectable by our assay (Table 2). At the time of sampling, NPWT with periodic instillation of Dakin’s solution was employed to provide local antifungal therapy to the subject’s bilateral lower extremity amputation sites, precluding effluent collection at these locations. Patient 1 survived to hospital discharge on post-injury day 193.

### Patient 2

**Figure 2:**
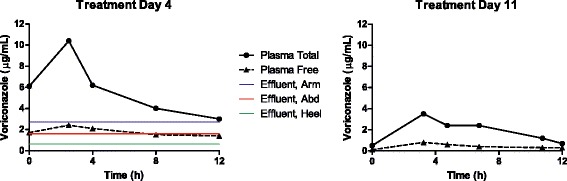
Voriconazole concentrations for Patient 2 in plasma and wound effluent on treatment day 4 (2a), and in plasma only on treatment day 11 (2b). Abd, abdomen.

A 28 year-old male was injured by a close-proximity blast from a rocket-propelled grenade, causing multiple penetrating shrapnel injuries to the chest and abdomen (including liver and duodenal lacerations), as well as fractures of the humerus and calcaneus. Following wound debridements and exploratory laparotomy with distal gastrectomy, duodenectomy, and abdominal fascia closure, NPWT devices were applied over surgical sites on the arm, abdomen, and heel. During routine wound debridements, *Aspergillus flavus* was cultured from soft tissues of the hip and posterior thigh on post-injury days 10, 13 and 14. On post-injury day 10, histopathology identified fungal elements in viable skeletal muscle tissue from the groin, and in non-viable connective tissues of the foot as well as non-viable serosal adhesions of the antrum of the stomach and first part of the duodenum. On post-injury day 21, fungal elements were observed again in necrotic tissues of the groin. No angioinvasion was observed, thus establishing the diagnosis as probable invasive fungal infection [9]. Plasma and wound effluent were sampled on post-injury day 15 (Patient 2a, Table 1, antifungal treatment day 4) following doses of L-AmB (5 mg/kg IV every 24 h) and voriconazole (4 mg/kg IV every 12 h). Concurrent medications potentially interfering with voriconazole metabolism given on this date included pantoprazole 40 mg IV every 12 h and quetiapine 25 mg PO every 24 h. Despite apparently adequate plasma levels of amphotericin B (trough 12.8 μg/mL, Figure 1) and voriconazole (trough 3.0 μg/mL, Figure 2), free concentrations of amphotericin B (unbound to protein) in wound effluent were below the limit of detection for the assay (<2.5 ng/mL, Table 2) and thus presumed to be sub-inhibitory for Mucorales (MIC ≤0.5 μg/mL). Whereas free voriconazole was detected at 2.7 μg/mL and 1.6 μg/mL from the arm and abdominal sites, respectively, the concentration in calcaneus effluent (0.6 μg/mL) was presumed to be sub inhibitory (MIC ≤1.0 μg/mL). In patient 2, plasma PK sampling was again performed for voriconazole only on post-injury day 21 (Patient 2b, Table 1, treatment day 11, and 7 days after the initial PK sampling). Concurrent medications potentially interfering with voriconazole metabolism given on this date included pantoprazole 40 mg IV every 12 h and ciprofloxacin 400 mg IV every 8 h. Notably, the systemic clearance of voriconazole increased nearly three-fold while the weight-adjusted volume of distribution more than doubled (Table 1). This likely reflected improvement in hepatic function indicated by decreasing aspartate aminotransferase and alanine transaminase values, as well as discontinuation of quetiapine 6 days previously. Pantoprazole was continued at the same dose and frequency at both sampling periods. These changes resulted in a significantly reduced 12-hour area under the curve (AUC), a measure of overall voriconazole exposure, and much lower peak and subtherapeutic trough concentrations. These changes occurred despite an increased mg/kg dose of voriconazole, which in turn resulted from catabolic loss of 17 kg over 1 week caused by limiting feeding in the setting of penetrating gastrointestinal trauma. Patient 2 survived to hospital discharge on post-injury day 99.

**Table 1:** **Patient characteristics and pharmacokinetic values**

Patient	1	2a		2b
Post-Injury Day	18	15		21
Treatment Day	15	4		11
Age (years)	30	28		28
Weight (kg)	78.8	77.1		60.1
Antifungal	L-AmB	L-AmB	Voriconazole	Voriconazole
Dose (mg)	400	400	320	320
Dose (mg/kg)	5.1 (24 h)	5.2 (24 h)	4.2 (12 h)	5.3 (12 h)
Peak (μg/mL)	25.5	58.4	10.4	3.5
Trough (μg/mL)	6.1	12.8	3.0	0.7
AUC_τ_(μg · h/mL)	262.5	534.7	67.5	24.1
T_1/2_ (h)	23.3	16.3	7.6	3.2
V_d_ (L/kg)	0.52	0.21	0.58	1.28
Clearance (mL/kg/min)	0.32	0.16	1.02	3.69
Plasma % bound	100%	100%	32.7 ± 8.2	22.1 ± 4.1%
Effluent % bound			55.6% (RUE)	N/A
	100%	100%	75.0% (abd.)	
	(all sites)	(all sites)	90.5% (calc.)	
eGFR (mL/min/1.73 m^2^)	19.9	75.8		126.1
AST/ALT (IU/L)	193/59	211/318		61/72
INR	1.4	1.4		1.2

Dose interval: 24 hours for L-AmB, 12 hours for voriconazole. Abd, abdomen; AST, aspartate aminotransferase; ALT, alanine aminotransferase; calc, calcaneus; eGFR, estimated glomerular filtration rate; INR, international normalized ratio; RUE, right upper extremity; T_1/2_, half-life; V_d_, volume of distribution.

**Table 2:** **Effluent antifungal concentration and negative-pressure wound therapy parameters**

				**Amphotericin B (μg/mL)**		**Voriconazole (μg/mL)**	
**Patient**	**Site**	**Pressure (mmHg)**	**Effluent (mL)**	**Total**	**Free**	**Total**	**Free**
1	Abd	−125	14	0.3	<LOD	--	--
	LLQ	−125	37	0.2	<LOD	--	--
2a	Arm	−125	96	3.0 ± 0.4	<LOD	2.7 ± 0.2	1.5 ± 0.002
	Abd	−125	60	1.3 ± 0.1	<LOD	1.6 ± 0.2	1.2 ± 0.01
	Heel	−125	28	1.3 ± 0.1	<LOD	0.6 ± 0.1	0.6 ± 0.01

LOD, limit of detection (2.5 ng/mL). Abd, abdomen; LLQ, left lower quadrant; LOD, limit of detection.

## Conclusions

Invasive fungal infection is a complication of combat injuries, particularly those sustained in southern Afghanistan, with risk factors of dismounted complex blast injury and high blood transfusion requirements during trauma resuscitation [6,25]. Necrotizing soft-tissue infections following natural disasters, such as tornados, have also been documented recently [7]. In an effort to optimize the care of severely injured and critically ill patients with fungal infections, we measured antifungal concentrations in the plasma of two patients. In addition, we determined antifungal concentrations in wound effluent to infer the passage of antifungal agent through and across the wounds under negative pressure. Interestingly, free concentrations of amphotericin (according to theory, the fraction available to exert antifungal activity) were undetectable in the wound effluent captured from all five sites in two patients with high concurrent plasma concentrations. In one of the patients, free voriconazole was found at concentrations likely sufficient to inhibit molds with an MIC ≤1.0 μg/mL in the effluent of two sites. However, concentrations were likely sub-inhibitory in effluent from a wound overlying the calcaneus, a site known to be challenging for adequate drug penetration due to its limited blood flow [26]. Consistent with the treatment of invasive fungal infection in immunocompromised populations, surgical debridement has been the primary therapy, with antifungal medications playing an adjunctive role. This is highlighted by the observation that Patient 1 recovered without receiving voriconazole despite the occurrence of *Aspergillus terreus*, a species with intrinsically reduced susceptibility to amphotericin B.

We observed significant variation in plasma PK parameters for L-AmB between the two patients who had a nearly 2-fold increase in the AUC despite identical age and body weight, notwithstanding bilateral lower extremity amputations in Subject 1 (the impact of amputations on systemic PK is undefined). This PK variability may reflect differences in renal excretion as Subject 1 had renal insufficiency at the time of sampling (Table 1). Likewise, the two patients may have had non-quantifiable differences in their capacity for hepatic metabolism of L-AmB given their differential elevation in aminotransferase levels (Table 1) [27]. Patient 2 demonstrated significantly increased voriconazole clearance between treatment days 4 and 11, as demonstrated by trough concentrations of 3.0 and 0.7 μg/mL, respectively. This may reflect, in part, improvements in his capacity for hepatic metabolism through the 2C19, 3A4 and 2C9 cytochrome P450 (CYP) enzymes, which constitute the major metabolic pathways for voriconazole [28,29]. A changing landscape of drug interactions may also have contributed, as quetiapine is a major substrate for CYP3A4 and minor substrate for CYP2D6, which are both minor metabolic pathways for voriconazole. Pantoprazole, an inhibitor of the enzyme primarily responsible for voriconazole metabolism (CYP2C19), was continued at the same dose during both sampling episodes, and thus was unlikely to be the sole contributor to the changes in voriconazole exposure. Ciprofloxacin is a weak inhibitor of CYP3A4, a minor metabolic pathway for voriconazole.

These PK parameter changes developed despite an increase in his mg/kg dose, attributable to significant weight loss while continuing to receive a fixed dose of voriconazole. Had the effluent been sampled again on treatment day 11, subtherapeutic voriconazole concentrations would likely have been found despite his dose exceeding the package-insert recommended dose of 4 mg/kg every 12 h. Furthermore, if this functional underdosing could have been detected via real-time therapeutic drug monitoring, a dose increase suitable for his demonstrated metabolic capacity could have been recommended. This situation highlights the need for vigilance of patients’ daily weights, and the potential need for frequent adjustments to medication dosages according to body weight, which can fluctuate significantly in both directions and for a variety of reasons in critically ill patients. Additionally, the significant degree of between-patient and within-patient PK variability we observed suggests that individuals requiring antimicrobial therapy, particularly for life-threatening infections, may benefit from real-time pharmacokinetic monitoring to optimize efficacy and reduce toxicity [30]. Unfortunately such monitoring is not clinically available for the majority of antimicrobial agents, and advancements in technology are needed to determine drug concentrations in real-time, ideally at or near the point of care.

This study has inherent limitations. Only two patients with 5 wounds were studied, reflecting limitations in the available patient population at our facility. In Patient 1, wound effluent could not be sampled from the histopathologically proven site of invasive fungal infection at the extremity amputation sites. Our conclusion that free amphotericin B levels were inadequate in the wound is based upon levels of protein binding in the wound effluent, outside the actual wound tissue, and we acknowledge that the degree of protein binding in effluent may not accurately reflect what is occurring in the wound tissue itself. However, free voriconazole was detectable in wound effluent, as we have also observed for numerous conventional antibacterial agents (unpublished data), and wound effluent cytokine analyses are currently being used to direct clinical care [31]. Our concern is also predicated on the canonical theory that the protein-bound fraction of an antimicrobial is inert against microorganisms [32]. Our investigation of effluent levels to reflect wound penetration is based upon practical limitations stemming from the requirement for an FDA Investigational Device Exemption to sample interstitial fluid concentrations using tissue microdialysis (the only such catheter currently approved by the FDA for human use is indicated for intracranial placement). Finally, although multiple fungal species were reported in the clinical record, neither antifungal MICs nor the isolates were available to confirm pathogen identity or antifungal susceptibility.

This preliminary report raises concern for the limited passage of unbound amphotericin B across wounds in combat casualties with proven or probable wound infections. These initial data require corroboration across larger data sets to ensure their clinical validity. Preclinical models and broader human sampling may prove useful to further characterize the penetration of currently available antifungal agents across infected tissues of combat wounds. Additionally, the PK changes we observed between patients and dynamic changes within one patient demonstrate a need for PK monitoring to optimize care for infections. Insights gained from such studies may lead to strategies to improve the efficacy of antifungals against invasive fungal infections.

### Consent

Written informed consent was obtained from the patients for publication of the Case report and any accompanying images. A copy of the written consent is available for review by the Editor of this journal.

## Abbreviations

ALT: Alanine transaminase
AST: Aspartate aminotransferase
AUC: Area under the curve
FDA: Food and drug administration
HPLC: High performance liquid chromatography
IV: Intravenous
L-AmB: liposomal amphotericin B
LRMC: Landstuhl Regional Medical Center
MIC: Minimum inhibitory concentration
NPWT: Negative pressure wound therapy
PK: Pharmacokinetics
